# Clinical pathway improves medical practice in total knee arthroplasty

**DOI:** 10.1371/journal.pone.0232881

**Published:** 2020-05-07

**Authors:** Noel Oizerovici Foni, Lauro Augusto Veloso Costa, Isabela Dias Paião, Isadora Orlando de Oliveira, Rogério Teixeira de Carvalho, Mario Lenza, Eliane Antonioli, Mario Ferretti

**Affiliations:** Hospital Israelita Albert Einstein, São Paulo, SP, Brazil; University of Rome 'La Sapienza', ITALY

## Abstract

**Purpose:**

Clinical pathways in total knee arthroplasty (TKA) consist of general guidelines, including several topics as early rehabilitation and antibiotic systematization, which are used to improve patient’s management, decrease complication rates and enhance clinical outcomes. The primary purpose of this study was to assess whether the use of a clinical pathway for TKA can contribute to reduce LOS and healthcare costs in a private hospital, without an increase in the hospital readmission rate. We also aimed to assess whether care providers adhered to the recommendations mainly antibiotic use and physical therapy.

**Methods:**

Retrospective cohort study of 485 patients who underwent TKA at private hospital. Patients were analyzed in two groups: Group I (GI), composed by 220 TKA patients, prior to the clinical pathway implementation, and Group 2 (GII), with 265 TKA patients post-clinical pathway. Several outcomes were analyzed: length of hospital stay, time from use of prophylactic antibiotic therapy, readmission within 30 days, physical therapy and costs associated to procedures and hospitalization rates.

**Results:**

The implementation of the clinical pathway was related with the reduction of the length of hospital stay from 6.3 days to 4.9 days (p = 0.021) without increase in readmissions. The physical therapy on the first postoperative day was most frequent in GII than GI (96.2% vs 78.1%, p < 0.001). Prophylactic ATB 60 minutes prior the surgery was significantly more used in GII than GI (99.2% vs 87.4%, p < 0.001). In addition, ATB suspension within 48 hours was significantly more frequent in GII than GI (84.7% vs. 51.6%, p < 0.001). The cost procedure of TKA showed a reduction of US$1,252.00 in GII when compared with GI (p<0,001).

**Conclusion:**

The implementation of a clinical pathway, with focus on early rehabilitation, for patients underwent TKA, contributed to a reduction of LOS and costs during hospital stay, with no increase in the readmission rate. We also concluded that there was adherence to the clinical pathway by care providers in our institution.

## Introduction

Total knee arthroplasty (TKA) is an intervention indicated as treatment for individuals in advanced stages of osteoarthritis, aiming to reduce pain and improve motor function.[1] Its demand has increased and is expected to impact long-term healthcare system costs. In the United States, 670,000 TKA were performed in 2012 at a total cost of 36.1 billion dollars, and due to high rates of primary TKA interventions, number of revisions TKA are also expected to grow 137% from 2005 to 2030. [1,2]

Clinical pathways are as an alternative by which health care efficiency can be achieved. Defined by a structured plan that indicates the sequence of events and actions necessary to achieve better outcomes, it involves the use of clinical protocols to improve management, decrease complication rates and optimize clinical outcomes. [3,4] It has been shown that the use of clinical pathway for total joint replacement results in the reduction of hospital length of stay (LOS), which is related to early rehabilitation protocols, resulting in fewer postoperative complications and without increase of the readmission rate. [2,4]

Recent studies have also shown that, in addition to clinical improvements, the interventions through a clinical pathway could help the healthcare system as a whole to reduce costs.[2,5] Although most clinical pathway for TKA has been implemented in the public health care system, rather than private hospitals, they could benefit from its use, as it is an organizational engagement process, which can add value for health insurance providers. However, there is still no consensus on the effectiveness of clinical pathways for TKA, since some reports have shown conflicting results regarding to efficacy of earlier physical therapy and discharge. [6,7]

The primary purpose of this study was to assess whether the use of a clinical pathway for TKA can contribute to reduce LOS and healthcare costs in a private hospital, without an increase in the hospital readmission rate. We also aimed to assess whether care providers adhered to the recommendations regarding, mainly antibiotic use and physical therapy. We hypothesize that standardization of care process has potential to improve these results.

## Methods

### Study design

Ethics and Research Committee of the Hospital Israelita Albert Einstein (number CAAE 57913016.4.0000.0071) approved the study. All data were analyzed anonymously so that there was no personal identification of the patients included in the registry. Because of this, there was no need for written informed consent from the participants. For data collection, we performed a retrospective analysis between 2009 and 2015 of the TKA registry at our institution, a private healthcare institution located in Brazil. Patient’s medical record was also analyzed to collect information required. A single investigator obtained all information. All patients who underwent primary TKA in that period were included in the study. Revision surgeries were excluded.

We designed a case-control study composed by two groups. Group I (GI) was composed of patients underwent TKA prior to the clinical pathway implementation. Group II (GII) consisted of patients post-clinical pathway.

### Clinical pathway’s development

A multidisciplinary team of healthcare professionals, which include surgeons, nurses and physical therapists, developed a clinical pathway to be used in cases of TKA. The general guidelines include recommendations that should be strictly followed during preoperative, intraoperative and postoperative period. The main items of the clinical pathway will be described.

The patient should perform immediate postoperative in the orthopedic unit, and if necessary an intensive care unit could be used. Use of compression socks is mandatory (use of a pneumatic compressor is optional). Pharmacologic thromboembolic prophylaxis is mandatory, but without specific guidelines of the medication type. The use of drain is optional (depending on the exudate flow rate it can be maintained until the second postoperative day).

Regarding rehabilitation, physical therapy should begin on the first postoperative day. The use of continuous passive motion machine (CPM) to gain range of motion (ROM) is available, but it is not mandatory. The onset of gait with progressive weight bearing is mandatory on the first postoperative day; it begins with gait training with walker or crutches. Discharge criteria includes ROM up to 90° and walking within a short/medium distance outside the room.

Antibiotic (ATB) prophylaxis must be done within 60 minutes before the incision and its use should be discontinued after 48 hours after surgery.

### Data assessment

The demographic variables were compared between two groups (GI and GII) in order to demonstrate the homogeneity among them: age, gender, body mass index (BMI) and comorbidity. Also, main recommendations of the clinical pathway about antibiotic use and physical therapy were analyzed and compared between the two groups in order to evaluate if there was adherence by care providers.

Rehabilitation data include time to onset of motor physical therapy and time to onset of gait. Regarding the use of ATB use, the time from ATB to incision and time until ATB suspension were measured. We obtained the cost of each procedure from the hospital cost center. The LOS was defined by the number of nights in the hospital. Composition of the cost includes not only the sum of daily hospitalization services, medications, exams, nursing and physiotherapy services, but also the cost of the prosthetic implant updated for the year of 2018. Incremental costs such as physician fees were not included in this analysis. The conversion from local currency in Real (R$) to Dollar (US$) was performed according to rates present in the study period (1US$ = 3,25R$).

Finally, to demonstrate that the implementation of the clinical pathway did not result in an increase of complications, we analyzed readmission rate within 30 days in both groups.

### Statistical analysis

Comparisons between two groups were performed using chi-square tests or Fisher's exact tests when considering categorical variables, and Student's t-tests or non-parametric Mann-Whitney tests when using quantitative variables. To investigate association of total cost of surgical procedure with observation period, a generalized linear model with gamma-distribution was adjusted. The results were presented by means of estimated and 95% confidence intervals (95% CI). The statistical analysis was performed using SPSS software. The significance level adopted was p < 0.05.

## Results

We analyzed data of 485 primary TKA, 220 patients in GI and 265 patients in GII. There were no significant differences between two groups regarding gender, age, BMI and comorbidity. There was a higher prevalence of women in both groups (72.3% in GI and 69.8% in GII). Overall mean age was 69.8 years (69.8 in GI and 69.9 in GII). The mean of BMI was 29.1 in GI and 28.7 in GII. The most frequent comorbidity observed in both groups was systemic arterial hypertension (56.8% in GI and 50.6% in GII) followed by diabetes (16.8% in GI and 23.4% in GII). Compression socks were used in all cases in postoperative period, and most of them used pneumatic compressor (98.6% in GI and 98.9% in GII). The most widely used drug for thromboprophylaxis was enoxaparin in both groups. Use of surgical drains was frequent in both groups (98.2% in GI and 91.3% in GII), and withdrawal occurred most often within 48 hours (95.0% in GI and 89.1% in GII).

We observed there was adherence by care providers to recommendations regarding physical therapy and prophylactic ATB usage (Table 1). The physical therapy on the first postoperative day was most frequent in GII than GI (96.2% vs 78.1%, p < 0.001). There was also difference between groups in relation to onset of gait. The median for patients in GI was 3 (2–4) days, and for patients in GII was 2 (2–3) days (Table 1). Prophylactic ATB 60 minutes prior the surgery was significantly more used in GII than GI (99.2% vs 87.4%, p < 0.001). In addition, ATB suspension within 48 hours was significantly more frequent in GII than GI (84.7% vs. 51.6%, p < 0.001).

**Table 1:** Comparison between GI and GII regarding main items of the clinical pathway.

		Total (n = 485)	GI (n = 220)	GII (n = 265)	
**ATB prior to 60 minutes to incision**^a^	Yes	448 (93.9%)	188 (87.4%)	260 (99.2%)	**p < 0.001**
	No	29 (6.1%)	27 (12.6%)	2 (0.8%)	
**ATB suspension within 48 hours**^**b**^	Yes	272 (68.0%)	95 (51.6%)	183 (84.7%)	**p < 0.001**
	No	128 (32.0%)	89 (48.4%)	33 (15.3%)	
**Time to onset of physical therapy (days)**	1	427 (88%)	172 (78.1%)	255 (96.2%)	**p < 0.001**
	2	53 (11%)	43 (19.5%)	10 (3.8%)	
	3	5 (1.0%)	5 (2.4%)	0 (0.0%)	
**Time to onset of gait (days)**^§^	Median	2 (2–3)	3 (2–4)	2 (2–3)	**p < 0.001**
	Min-max	1–11	1–11	1–7	
**30-day readmission**	No	482 (99.4%)	217 (98.6%)	265 (100.0%)	**p = 0.093**
	Yes	3 (0.6%)	3 (1.4%)	0 (0.0%)	

a: 8 patients excluded of this analysis for insufficient data

b: 85 patients excluded of this analysis for insufficient data

§: 16 patients excluded of this analysis for insufficient data.

During the time period of the study, we observed that only 1.4% of the patients were re-admitted for up to 30 days in GI and no cases were reported in GII.

### Length of stay

**Fig 1:**
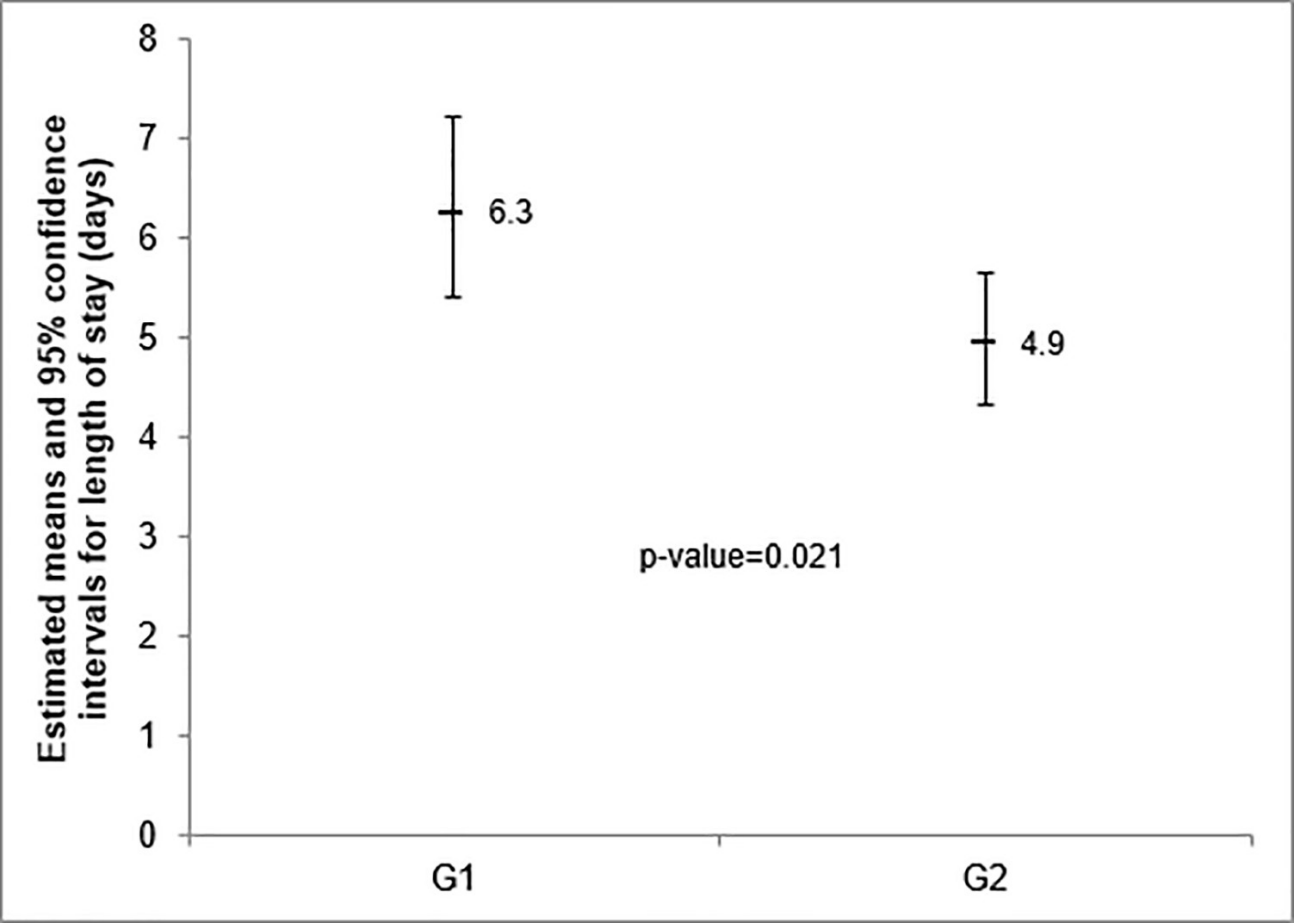
Length of stay.

We observed that there was a significant reduction in the LOS after implementation of clinical pathway. The median for patients in GII was 4.9 (4.3–5.6) days, and for patients in GI was 6.3 (5.4–7.2) days (p < 0.05). For this analysis, we excluded 3 patients in the GI and 3 patients in the GII who had length of stay greater than 15 days due complications not related to TKA procedure (Fig 1).

### Cost analysis

**Fig 2:**
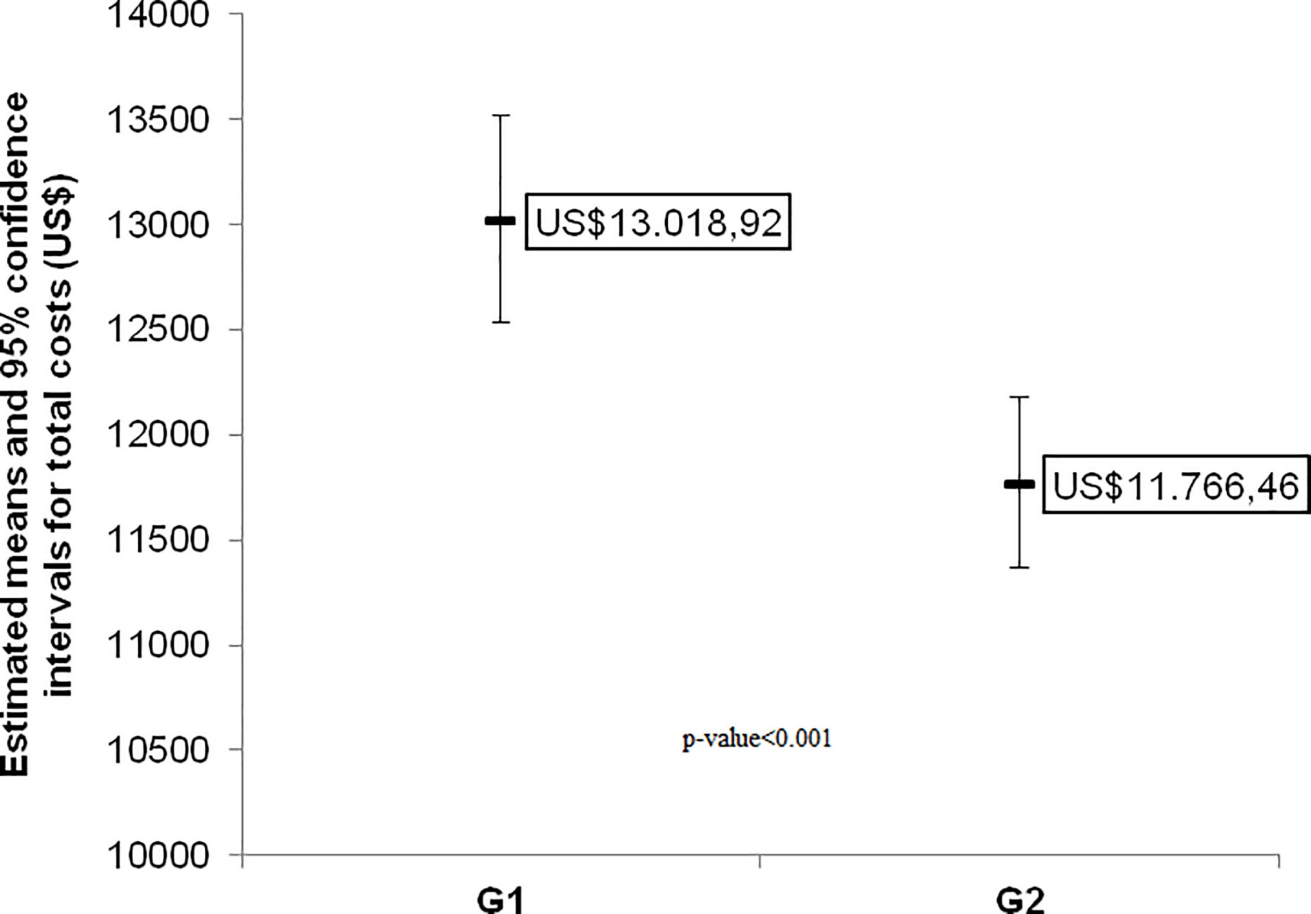
Total cost of the surgical procedure.

The cost analysis showed a reduction in surgical procedure costs after clinical pathway implantation, from US$ 13,018.92 (12,538.48–13,517.76) in GI to US$11,766.46 (11,370.52–12,176.23) in GII (p <0.001). For this analysis, four patients (2 from GI and 2 from GII) were excluded because total cost included treatment of the other complications not related to TKA. Overall, there was a cost reduction of US$1,252.00 in GII when compared with GI (Fig 2).

## Discussion

This present study has shown that our clinical pathway was able to reduce costs and hospital LOS of TKA patients, without increasing readmission rate. Also, we showed that there was adherence by care providers to recommendations regarding antibiotic use and physical therapy. Our findings are in line with previous studies, showing that standardization of patient’s care process was able to improve medical practice. [3,5,6,8,9]

One of main purposes of clinical pathway is to allow early but safely hospital discharge of the patient, since keeping patients in hospital could increase not only postoperative complications but also direct hospital cost. [5,8] In addition, there is direct association between LOS and patient’s satisfaction.[6,10] However, to reach purpose of reducing LOS is not such simple as many patient characteristics are associated with it, like age, sex, marital status, co-morbidity and early rehabilitation.[6,11] Clinical pathway at our institution focused on early rehabilitation and we believe that this factor was the primarily responsible for reducing LOS. We showed there was a significant increase in the number of patients starting physical therapy within 24 hours of surgery. For being a modifiable factor, accelerated rehabilitation with early physical therapy also is key component of the most clinical pathways and other perioperative protocols with the same purposes.[12]. Tayrose et al. [13] in a study with nine hundred hip and knee arthroplasties, have shown that patients beginning physical therapy still in the recovery room had significantly less LOS than patients who began physical therapy on the first postoperative day. Similar to our results, Ayalon et al. [3] showed that a clinical pathway focused on faster rehabilitation, with onset of range of motion on the same day of surgery, was able to reduce the average LOS from 4.03 to 3.77 days. They also showed there was no significant increase in complications. Our results are also in agreement with results reported by Hertog et al. [9], who reduced LOS was accompanied by fewer adverse events in the group of patients underwent physical therapy on the same day as the TKA surgery.

Besides patient-related factors, as already mentioned, there are also structural-related risk factors influencing LOS.[11] Piuzzi et al. [11] have reported patient-related factors such as sex, age and comorbidities are important but are not the main drivers of the LOS. Our results corroborate with their results, since the two groups did not differ regarding demographic characteristics despite difference found in the LOS. Although our clinical pathway focuses on early rehabilitation, other factors are also important, such as patient education, pain management and anesthesia care.[3,4,14] Ayalon et al.[3] have reported results similar to ours, with a clinical pathway focusing on not only early rehabilitation, but also on better pain and nausea management, standardization of anesthesia care and patient education. These findings highlight the importance of the clinical pathway as a tool to be developed on the health care institutions with help of multidisciplinary team for improving patient outcomes and reducing costs.

There seems to be a clear interaction between the LOS and overall costs of TKA. Barbieri et al. [8], performed a meta-analysis to evaluate clinical pathway for TKA compared with standard medical care. They reported lower costs during hospital stay associated with use of clinical pathway. Also, a shorter LOS was observed in the clinical pathway group. Similar results were reported by Lovald et al. [15] They performed a study comparing outpatient surgery with patients who stayed 3–4 days in the hospital. Costs associated with outpatient procedure, after two years, were US$ 8,527 lower than 3–4 days group. In our study, we observed a cost reduction of almost US$2,000 per surgery during hospital stay, with no increase in readmission rate probably due to decrease in LOS and better medical practice. By standardizing the care process and decreasing LOS without an increase in postoperative complications, we find that adoption of a clinical pathway can become an important strategy in reducing costs of TKA. These benefits reached with clinical pathway, as cost and LOS reduction, are still more important considering setting of a developing country. Difficulties in health care system in many of these countries are even greater and a tool as clinical pathway could help by setting guidelines to be adopted by surgeons. In addition, cost of this procedure is comparable with developed countries, and since the budget of our health system is increasingly limited, there is a growing need for cost reduction programs, by the demand of health system.[16] We did not have cost date for comparison between public and private setting. There is a shortage of Brazilian arthroplasty data in the literature. Ferreira et al. [17] showed date on public care but without data about costs. They have shown the reduction in LOS from 2008 to 2015 in Brazil, being the Midwest region of the country with the highest average of reduction (6.6 days), showing the heterogeneity of health care system in that country.[17]

This study has some limitations. First, data were collected retrospectively, which leaves them prone to information bias. Second, we cannot quantify the real importance of each item of the clinical pathway in the analyzed outcomes. Third, our study was performed in a private hospital, and this may have implications on the generalizability of our findings. Finally, we do not have neither clinical outcomes nor the cost of the development and implementation of the clinical pathway, which makes it impossible to evaluate if all this process is cost-effectiveness. Despite these limitations, our findings suggest that clinical pathways, when they are well structured, can be safely implemented and actively used in TKA patients.

## Conclusion

The implementation of a clinical pathway, with focus on early rehabilitation, for patients underwent TKA, contributed to a reduction of LOS and costs during hospital stay, with no increase in the readmission rate. We also concluded that there was adherence to the clinical pathway by care providers in our institution.

## List of abbreviations

TKA: Total Knee Arthroplasty
CP: Clinical Pathway
PO: Postoperative
ATB: Antibiotic
LOS: Length of Stay
THA: Total Hip Arthroplasty
